# Seafloor doming driven by degassing processes unveils sprouting volcanism in coastal areas

**DOI:** 10.1038/srep22448

**Published:** 2016-03-01

**Authors:** Salvatore Passaro, Stella Tamburrino, Mattia Vallefuoco, Franco Tassi, Orlando Vaselli, Luciano Giannini, Giovanni Chiodini, Stefano Caliro, Marco Sacchi, Andrea Luca Rizzo, Guido Ventura

**Affiliations:** 1 Istituto per l’Ambiente Marino Costiero, Consiglio Nazionale delle Ricerche, Naples, Italy; 2 Dipartimento di Scienze della Terra, Università di Firenze, Florence, Italy; 3 Istituto di Geoscienze e Georisorse, Consiglio Nazionale delle Ricerche, Florence, Italy; 4 Istituto Nazionale di Geofisica e Vulcanologia, Bologna, Italy; 5 Istituto Nazionale di Geofisica e Vulcanologia - Osservatorio Vesuviano, Naples, Italy; 6 Istituto Nazionale di Geofisica e Vulcanologia, Palermo, Italy; 7 Istituto Nazionale di Geofisica e Vulcanologia, Roma, Italy

## Abstract

We report evidences of active seabed doming and gas discharge few kilometers offshore from the Naples harbor (Italy). Pockmarks, mounds, and craters characterize the seabed. These morphologies represent the top of shallow crustal structures including pagodas, faults and folds affecting the present-day seabed. They record upraise, pressurization, and release of He and CO_2_ from mantle melts and decarbonation reactions of crustal rocks. These gases are likely similar to those that feed the hydrothermal systems of the Ischia, Campi Flegrei and Somma-Vesuvius active volcanoes, suggesting the occurrence of a mantle source variously mixed to crustal fluids beneath the Gulf of Naples. The seafloor swelling and breaching by gas upraising and pressurization processes require overpressures in the order of 2–3 MPa. Seabed doming, faulting, and gas discharge are manifestations of non-volcanic unrests potentially preluding submarine eruptions and/or hydrothermal explosions.

Deep-sea hydrothermal fluid (thermal waters and gases) discharges are a common feature of mid-ocean ridges and convergent plate margins including the submerged portion of island arcs, whereas cold emissions of gas hydrates (chlatrates) generally characterize continental shelves and passive margins^1^,^2^,^3^,^4^,^5^. The occurrence of submarine hydrothermal discharges in coastal areas implies a heat source (magma reservoir) within the continental crust and/or the mantle. These discharges may precede magma upraising through the uppermost level of the crust and, ultimately, eruptions and emplacement of volcanic seamounts^6^. Therefore, the recognition of (a) morphologies related to active deformation of the seabed and (b) gas emissions near densely inhabited coastal areas like the Neapolitan volcanic area in Italy (about 1 million inhabitants) is of primary importance for an evaluation of possible volcanic eruptions at shallow depth. In addition, while the morphological features associated with deep-sea hydrothermal or hydrate gas emissions are relatively well known^7^,^8^,^9^,^10^,^11^ because of their geological and biological peculiarities, those associated with shallower waters are, with the exception of those occurring in lakes^12^, comparatively less documented. Here, we present new bathymetric, seismic, water column and geochemical data on a submerged, morphologically and structurally complex area affected by gas emissions located in the Gulf of Naples (southern Italy), about 5 km offshore from the Naples harbor. These data have been collected during the SAFE_2014 (August 2014) cruise performed on board of R/V Urania. We describe and interpret the seafloor and subsurface structures where the gas discharges occur, investigate the origin of the discharged fluids, identify and characterize the mechanisms that regulate the gas upraising and the associated deformations, and discuss the volcanological implications.

The Gulf of Naples forms the western margin of the Plio-Quaternary, NW-SE elongated Campanian Plain structural depression^13^,^14^,^15^. The gulf is confined to the north by the E-W arranged, active volcanoes of Ischia Island (from about 150 ka to 1302 AD), Campi Flegrei caldera (from about 300 ka to 1538 AD), and Somma-Vesuvius (from <360 ka to 1944 AD)^15^, while to the south it is bordered by the Sorrento Peninsula (Fig. 1a). The Gulf of Naples is affected by prevailing NE-SW and second-order NW-SE striking faults (Fig. 1)^14^,^15^. Ischia, Campi Flegrei and Somma-Vesuvius are characterized by hydrothermal manifestations, ground deformation, and shallow seismicity^16^,^17^,^18^ (e.g., the 1982–1984 unrest episode of Campi Flegrei with an uplift of 1.8 m and thousands of earthquakes). Recent studies^19^,^20^ suggest a possible link between the dynamics of Somma-Vesuvius and that of Campi Flegrei, possibly related to a ‘deep’, single magma reservoir. The volcanic activity in the last 36 ka at Campi Flegrei and 18 ka at Somma Vesuvius, and the sea level oscillations have controlled the depositional system in the Gulf of Naples^21^. A lowstand of the sea level in the last glacial maximum (18 ka) produced a regression of the paralic-shallow depositional systems with a later filling by transgressive episodes during the Late Pleistocene-Holocene. Submarine gas emissions have been recognized around Ischia Island and offshore near the coast of Campi Flegrei and Somma-Vesuvius (Fig. 1b).

## Results

**Figure 1:**
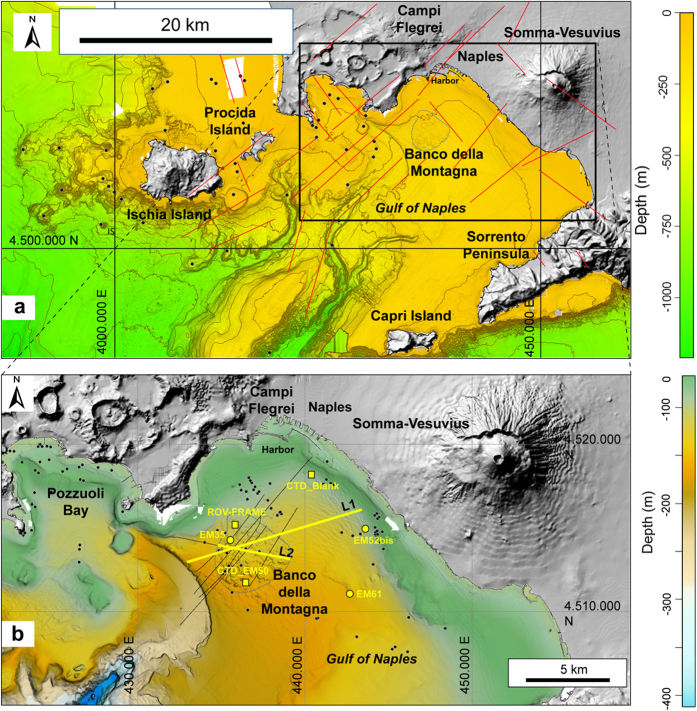
(**a**) Morphological and structural arrangement of the continental shelf and Gulf of Naples^15^,^23^,^24^,^48^. Dots are the main submarine eruptive centers; the red lines represent the main faults. (**b**) Bathymetry of the Gulf of Naples with the detected fluid vents (dots) and the trace of the seismic lines (black lines). Yellow lines are the traces of the seismic lines L1 and L2 reported in Fig. 6. The boundary of the Banco della Montagna (BdM) dome-like structure is marked by a bleu dashed line in (**a**,**b**). Yellow squares mark the location of the acoustic water column profile, CTD-EMBlank, CTD-EM50, and ROV frame reported in Fig. 5. Yellow circles mark the location of the sampled gas discharges, whose composition is reported in Table S1. Figure generated with Surfer® 13 by Golden Software (http://www.goldensoftware.com/products/surfer).

**Figure 2:**
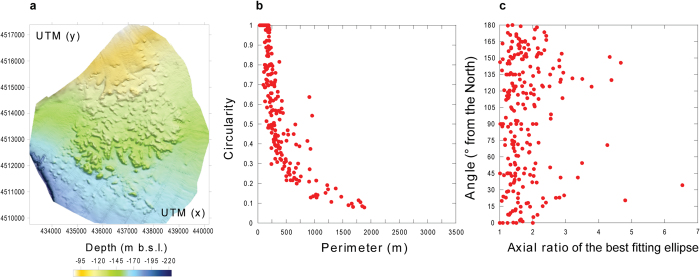
(**a**) Digital Terrain Model (1 m cell size) of the Banco della Montagna (BdM) dome. (**b**) Perimeter vs. Circularity of the BdM mounds. (**c**) Axial ratio vs. angle (orientation) of the major axis of the best fitting ellipses encircling the mounds. The standard error of the Digital Terrain model is 0.004 m; the standard errors of Perimeter and Circularity are 4.83 m and 0.01, respectively, and those of Axial ratio and angle are 0.04 and 3.34°.

**Figure 3:**
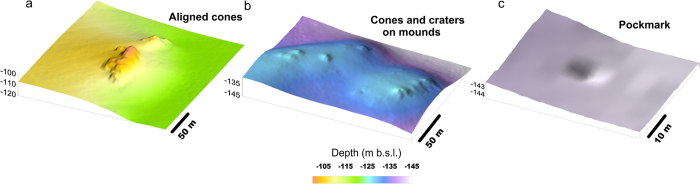
Details of the cones, craters, mounds, and pockmarks recognized in th BdM area extracted from the DTM of Fig. 2. (**a**) Aligned cones of a flat seabed; (**b**) Cones and craters emplaced on NW-SE elongated mounds; (**c**) pockmark emplaced on a gently dipping surface.

**Figure 4:**
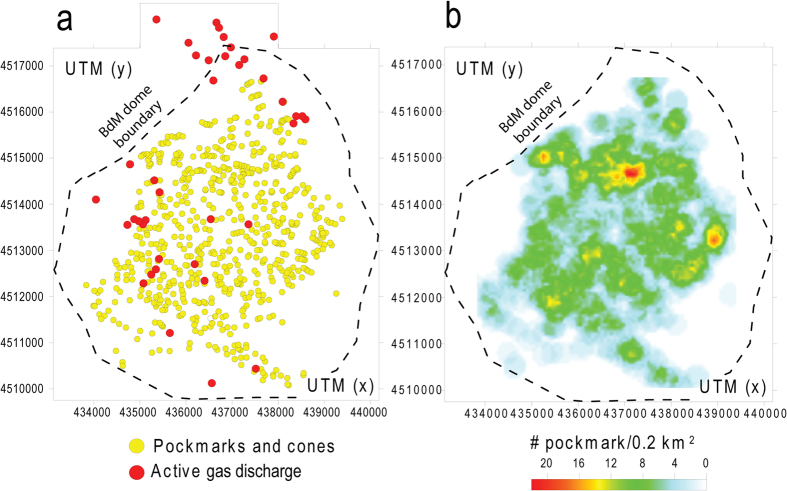
(**a**) Spatial distribution of the detected craters, pockmarks and active gas discharges. (**b**) Spatial density (number/0.2 km2) of the craters and pockmarks reported in (**a**).

A new Digital Terrain Model (DTM) with 1 m resolution of Gulf of Naples has been constructed on the basis of data acquired during the SAFE_2014 (August 2014) cruise (see Methods). The DTM shows that the seafloor south of the harbor of Naples is characterized by a southward facing, gently dipping (slope ≤3°) surface interrupted by a 5.0 × 5.3 km dome-like structure, locally known as Banco della Montagna (BdM) (Fig. 1a,b). BdM develops between about 100 and 170 m depth and it is 15–20 m higher than the surrounding seafloor. The BdM dome shows a hummocky morphology due to 280 sub-circular to elliptical mounds (Fig. 2a), 665 cones, and 30 pockmarks (Figs 3 and 4). The maximum height and perimeter of the mounds is 22 m and 1,800 m, respectively. The circularity [*C* = 4π(area/perimeter^2^)] of the mounds decreases as the perimeter increases (Fig. 2b). The axial ratio of the mounds ranges between 1 and 6.5, and those with axial ratio >2 show a preferred N45°E + 15° strike and a second-order, more dispersed, N105°E to N145°E strike (Fig. 2c). Single or aligned cones are present on the BdM flat surface and on the top of mounds (Fig. 3a,b). The cone alignments follow that of the mound on which they are emplaced. Pockmarks are prevalently located on the flat seabed (Fig. 3c) and, occasionally, on mounds. The spatial density of the cones and pockmarks evidences major NE-SW alignments delimiting the northeastern and southwestern boundary of the BdM dome (Fig. 4a,b); less extended NW-SE alignments are in the BdM central sector.

**Figure 5:**
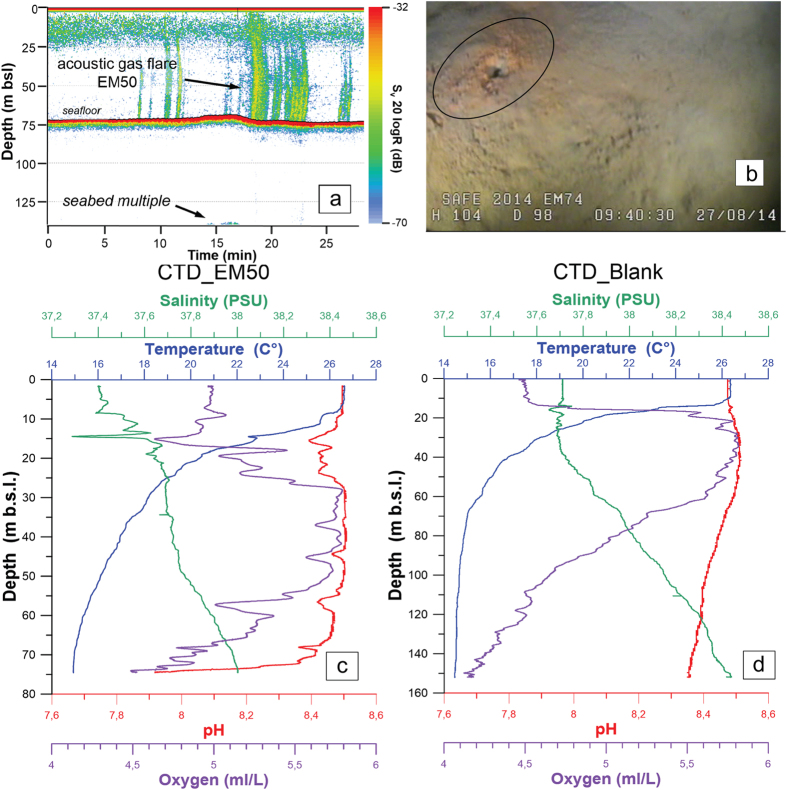
(**a**) Acquisition window of an acoustic water column profile (echosounder Simrad EK60). Vertical green bands corresponding to gas flares detected on the EM50 fluid emission (ca 75 m below sea level) located in the BdM area; seabed multiple signal on the bottom and seafloor are also shown (**b**) Single photo collected with a Remote Operated Vehicle in the BdM area showing a small crater (black circle) surrounded by red to orange sediments. (**c**,**d**) Multi-parameters probe CTD data processed with the SBED-Win32 software (Seasave, version 7.23.2). Patterns of selected parameters (Salinity, Temperature, pH and Oxygen) of the water column above the fluid emission EM50 (panel **c**) and outside the Bdm discharge area panel (**d**).

We recognize 37 gas emissions in the BdM area from echo-sounder images of the water column and direct observations on the sea bottom with ROV acquired during the SAFE_2014 cruise in August 2014 (Figs 4 and 5). The acoustic anomalies of these emissions show vertically elongated shapes upraising from the seafloor and a vertical extent between 12 and about 70 m (Fig. 5a). In some places, the acoustic anomalies form nearly continuous ‘trains’. The observed bubble plumes are highly variable: from a continuous, dense bubble-flux to short-lived phenomena (Supplementary Movie 1). ROV inspection allowed the visual verification of the occurrence of fluid vents from the seafloor and highlighted the presence of small pockmarks on the seabed, sometimes surrounded by red to orange colored sediments (Fig. 5b). In some cases, the ROV passage reactivated the emissions. Vent morphologies show a top circular opening without flares in the water column. The pH values in the water column just above the discharge sites show a significant drop indicating local more acidic conditions (Fig. 5c,d). In particular, the pH values above a BdM gas emission located at 75 m depth decrease from 8.4 (at 70 m depth) to 7.8 (at 75 m depth) (Fig. 5c), whereas others sites in the Gulf of Naples have pH values between 8.3 and 8.5 in the depth interval 0–160 m (Fig. 5d). Significant variations of the seawater temperature and salinity in two sites located inside and outside the BdM area in the Gulf of Naples are lacking. At 70 m depth, the temperature is 15 °C and the salinity is about 38 PSU (Fig. 5c,d). The measured values of pH, temperature and salinity indicate: a) the involvement of acid fluids associated to the BdM degassing process, and b) the absence, or very slow discharge, of hot fluids and brines.

**Figure 6:**
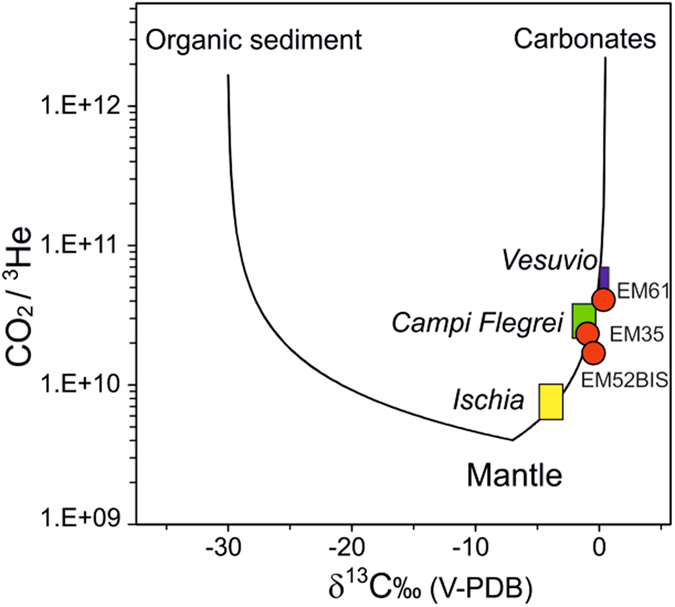
δ^13^C of CO_2_ vs. CO_2_/^3^He plot of the gas EM35, EM61 and EM52BIS (red dots, data in Table S1). The mixing lines between a mantle composition and the end-members of limestone and organic sediment^22^ are reported for comparison. The boxes represent the fields of the fumaroles of Ischia Island, Campi Flegrei, and Somma-Vesvius^59^,^60^,^61^. BdM samples are on the mixing trend of the Campanian volcanoes. The end-member gases of the mixing line are a mantle source and the gases produced by decarbonation reaction of carbonate minerals.

We collected three gas samples from the study area between 22 and 28 August 2014. These samples show a similar composition, largely dominated by CO_2_ (934–945 mmol/mol), followed by relevant concentrations of N_2_ (37–43 mmol/mol), CH_4_ (16–24 mmol/mol) and H_2_S (0.10–0.44 mmol/mol), whereas H_2_ and He are less abundant (<0.052 and <0.016 mmol/mol, respectively) (Fig. 1b; Table S1, Supplementary Movie 2). Relatively high concentration of O_2_ and Ar (up to 3.2 and 0.18 mmol/mol, respectively) were also measured. Light hydrocarbons, whose sum ranges from 0.24 to 0.30 mmol/mol, consist of C2–C4 alkanes, aromatics (mainly benzene), propene and S-bearing compounds (thiophenes). The ^40^Ar/^36^Ar values are consistent with that of air (295.5) although the value of the sample EM35 (BdM dome) is 304, showing a slight ^40^Ar-excess. The δ^15^N ratios are higher than that of air (up to +1.98% vs. Air), whereas the δ^13^C-CO_2_ values range from −0.93 to 0.44% vs. V-PDB. The R/Ra values (after correction for air contamination using the ^4^He/^20^Ne ratios) are between 1.66 and 1.94, indicating the presence of a significant fraction of mantle He. A further indication on the origin of the gas discharged in BdM is given by combining the helium isotopes with CO_2_ and its stable isotopes^22^. In the CO_2_/^3^He vs. δ^13^C of CO_2_ plot (Fig. 6), the BdM gas composition is compared with that of the fumaroles from Ischia Island, Campi Flegrei and Somma-Vesuvius. Figure 6 also reports the theoretical mixing lines among the three different carbon sources possibly involved in the generation of BdM gases: exsolving mantle-derived melts, organic matter-rich sediments, and carbonates. The BdM samples fall on the mixing line depicted by the three Campanian volcanoes, i.e. the mixing between mantle gases (here considered slightly more enriched in CO_2_ with respect to the classical MORB in order to fit the data) and those produced by decarbonation reactions of crustal rocks.

**Figure 7:**
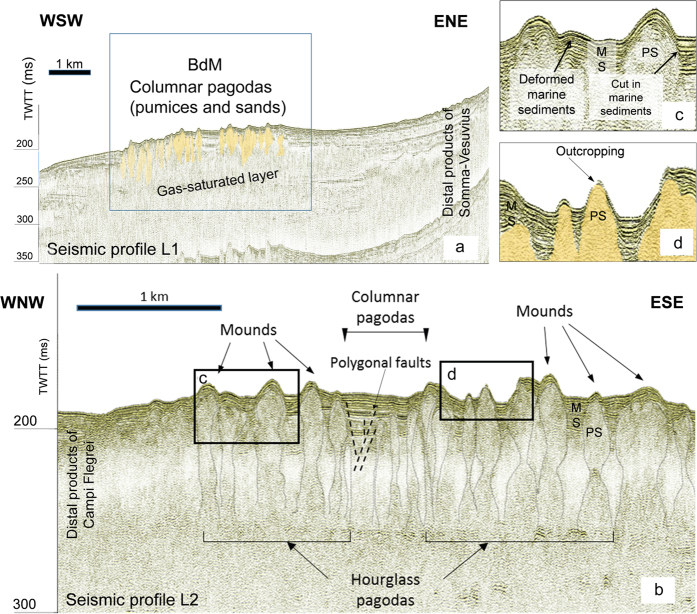
(**a**) Mono-channel seismic profile L1 (navigation track in Fig. 1b) showing the spatial arrangement of columnar shapes (pagodas). Pagodas are made up of chaotic sediments constituted by pumices and sands. The presence of a gas-saturated layer below the pagodas obliterates the continuity of the deeper stratigraphic layers. (**b**) Mono-channel seismic profile L2 (navigation track in Fig. 1b) with highlighted seafloor mounds, cut and deformations of the marine (MS) and pumice-sand sediments (PS). (**c**) Detail of the deformations in MS and PS are reported in (**c**,**d**). Assuming a velocity of 1580 m/s for the uppermost sediments, 100 ms represent about 80 m in the vertical scale.

Seismic profiles L1 and L2 (Figs 1b and 7) show the transition between BdM and the distal stratigraphic sequences of Somma-Vesuvius (L1, Fig. 7a) and Campi Flegrei (L2, Fig. 7b) volcanic areas. BdM is characterized by the presence of two main seismo-stratigraphic layers (MS and PS in Fig. 7). The uppermost one (MS) shows sub-parallel reflectors of high to medium amplitude and lateral continuity (Fig. 7b,c). This layer includes the post-Last Glacial Maximum (LGM) systems tract marine deposits, which consist of sands and clays^23^. The underlying PS layer (Fig. 7b–d) is characterized by chaotic to transparent facies and shows a columnar or hourglass shape. The top of the PS deposits form the seabed mounds (Fig. 7d). These diapir-like geometries testify the intrusion of the PS transparent material in the uppermost MS deposits. The upraising is responsible for the formation of folds and faults affecting the MS layers and the overlying, present-day sediments of the BdM sea bottom (Fig. 7b–d). MS stratigraphic interval is clearly stratified in the ENE portion of the L1 profile, while it is whitening toward BdM due to the presence of a Gas Saturated Layer (GSL) roofed by some inner levels of the MS sequence (Fig. 7a). Gravity cores collected at the top of BdM in correspondence of the transparent seismic layer show that the uppermost 40 cm consist of recent to presently depositing sands; the underlying succession is made up of these sands chaotically mixed with pumice fragments of the ‘Pomici Principali’ (dated 11.9–12.1 ka)^24^,^25^ and ‘Neapolitan Yellow Tuff’ (14.8 ka)^26^ explosive eruptions of Campi Flegrei. The transparent facies of the PS layer cannot be explained by chaotic mixing processes alone because the chaotic layers found away from BdM in Gulf of Naples and related to landslides, mud-flows and pyroclastic flows, are not acoustically transparent^21^,^23^,^24^. We conclude that the observed PS seismic facies of BdM, as well as the occurrence of outcropping PS layers on the sea bottom (Fig. 7d) reflects the uprising of gas.

## Discussion

The morphological and structural features of BdM are similar to those of other submarine hydrothermal and gas hydrate fields worldwide^2^,^12^,^27^,^28^,^29^,^30^,^31^,^32^,^33^,^34^ and commonly associated with the upraising (doming and mounds) and discharge (cones, pockmarks) of gas. The BdM aligned cones and pockmarks, and the elongated mounds suggest a structurally-controlled permeability (Figs 2 and 3). The spatial arrangement of the mounds, pockmarks, and active vents indicate that their distribution is partly controlled by NW-SE and NE-SW striking fractures (Fig. 4b). These are the preferred strikes of the fault systems affecting the Campi Flegrei and Somma-Vesuvius volcanic areas and Gulf of Naples. In particular, the former structures control the location of the hydrothermal discharge at the Campi Flegrei caldera^35^. Therefore, we conclude that the faults and fractures of the Gulf of Naples represent the preferred pathway for the gas migration to the surface, a feature common to other, structurally controlled hydrothermal systems^36^,^37^. It is noteworthy that the BdM cones and pockmarks are not always associated with mounds (Fig. 3a,c). This indicates that the mounds do not necessarily represent precursors to pockmark formation, as suggested by other authors for gas hydrate zones^32^,^33^. Our conclusion supports the hypothesis that the breaching of domed seafloor sediments does not always lead to the formation of pockmarks^34^.

The three collected gas discharges show chemical features typically recognized in hydrothermal fluids, i.e. predominance of CO_2_ and significant concentrations of reduced gases (H_2_S, CH_4_ and H_2_) and light hydrocarbons (especially benzene and propene)^38^,^39^,^40^,^41^,^42^,^43^,^44^,^45^ (Table S1). The occurrence of atmospheric gases, such as O_2_, whose presence is not expected to be occurring in submarine emissions, is likely due to contamination from air dissolved in seawater at the contact with the gases stored in the plastic box used for sampling as the ROV was uprising from the sea bottom to the surface. On the contrary, the positive δ^15^N value as well as the high N_2_/Ar (up to 480), which are significantly higher than that of ASW (Air Saturated Water), suggests that most N_2_ is produced by an extra-atmospheric source, in agreement with the dominant hydrothermal origin for these gases. The hydrothermal-volcanic origin of the BdM gas is confirmed by the CO_2_ and He contents and their isotopic signature. Carbon isotopes (δ^13^C-CO_2_ from −0.93% to +0.4%) and the CO_2_/^3^He values (from 1.7 × 10^10^ to 4.1 × 10^10^) show that the BdM samples belong to the mixing trend of the fumaroles from the volcanoes surrounding the Gulf of Naples between a mantle end-member and gases produced by decarbonation reactions (Fig. 6). More specifically, the BdM gas samples have roughly the same position along the mixing trend as the fluids from the adjacent Campi Flegrei and Somma-Veusivus volcanoes. They are more enriched in the crustal component than the Ischia fumaroles, which are closer to the mantle end-member. The value of ^3^He/^4^He is higher at Somma-Vesuvius and Campi Flegrei (R/Ra between 2.6 and 2.9) than at BdM (R/Ra between 1.66 and 1.96; Table S1). This suggests the addition and accumulation of radiogenic He originated from the same magmatic source feeding the Somma-Vesuvius and Campi Flegrei volcanoes. The absence of a detectable fraction of organic carbon in the BdM discharges indicates that the organic sediments are not involved in the BdM degassing process.

According to the above reported data and the results from experimental models on dome-like structures associated with submarine gas-rich areas^33^, gas pressurization at depth is likely responsible for the formation of the km-scale BdM dome. To have an estimate of the overpressure *P*_*def*_ responsible for the BdM doming, we applied a thin-plate mechanical model^33^,^34^ assuming that the BdM dome is, according to the collected morphological and seismic data, a sub-circular thin plate with a radius *a* larger than the vertical maximum displacement *w* and thickness *h* of the deformed soft cohesive sediments (Supplementary Figure S1). *P*_*def*_ represents the differences between the total pressure and the lithostatic pressure plus the pressure of the water column. At BdM, the radius is about 2,500 m, *w* is 20 m, and the maximum value of *h* estimated from the seismic profiles is in the order of 100 m. We calculate *P*_*def*_ from the relation^46^ *P*_*def*_ = *w* 64 *D*/*a*^4^, where *D* is the flexural rigidity; *D* is given by (*E h*^3^)/[12(1 – ν^2^)], where *E* is the Young’s modulus of the sediments and ν is Poisson’s ratio (~0.5)^33^. Since measurements of the mechanical properties on the BdM sediments are not available, we set *E* = 140 kPa, which is a reasonable value for coastal sand sediments^47^ like those of BdM^14^,^24^. We do not consider the higher *E*-values reported in the literature for silty clay sediments (300 < *E* < 350,000 kPa)^33^,^34^ because the BDM sediments mainly consist of sands and not of silt or silty clay^24^. We obtain *P*_*def*_ = 0.3 Pa, a value consistent with that estimated for seafloor doming processes in gas-hydrate basin settings^33^, where *P*_*def*_ varies between 10^−2^ to 10^3^ Pa, with the lower values representative of domes with low *w/a* and/or *h/a*. At BdM, a possible decrease in stiffness due to localized gas saturation of the sediments and/or the occurrence of pre-existing fractures could also promote failure and consequent gas release, thus allowing the formation of the observed venting structures. The collected reflection seismic profiles (Fig. 7) show that PS sediments upraise from GSL pushing up the overlain MS marine deposits, thus generating mounds, folding, faults and sedimentary cuts (Fig. 7b,c). This suggests that the 14.8 to 12 ka old pumices intrude the younger, MS layers by upward gas migration process. The morphological features of the BdM structures can be regarded as the result of the overpressure generated by fluid emissions arising from GSL. Given that active emissions are visible from the seafloor until more than 170 m b.s.l.^48^, we assume a fluid overpressure inside GSL of more than 1,700 kPa. The upward migration of gas within the sediments has also the effect to scrub the materials included in MS, thus explaining the presence of chaotic sediments in the gravity cores sampled over BdM^25^. In addition, a complex fracture system is spawned from the overpressure in GSL (the polygonal faults in Fig. 7b). Overall, this morphological, structural and stratigraphic settlement is known as ‘pagoda’^49^,^50^ and was initially attributed to secondary effects originated by old glacial morphologies, while it is currently interpreted as the effect of rising up of gas^31^,^33^ or evaporites^50^. In the Campanian continental margin evaporitic sediments are lacking, at least within the uppermost 3 km of the crust^51^. Therefore, the growth mechanism of the BdM pagodas is likely governed by the gas upraising within the sediment. This conclusion is supported by the transparent seismic facies of the pagodas (Fig. 7) and, as previously reported, by data on gravity cores^24^, in which the present-day sands are chaotically mixed with pumices of the ‘Pomici Principali’^25^ and ‘Neapolitan Yellow Tuff’^26^ eruptions of Campi Flegrei. In addition, the PS sediments intrude and deform the uppermost MS layers (Fig. 7d). This structural arrangement indicates that the pagodas represent uprising structures and not only gas conduits. As a result, two main processes control the formation of pagodas: a) a decrease in the density of the soft sediments as gas enters from below and b) the uprising of gas-sediment mixtures, which are responsible for observed folding, faulting, and breaking of the MS sediments (Fig. 7). A similar mechanism of formation as been proposed for the pagodas associated to gas-hydrate in the Southern Scotia Sea (Antarctica)^31^. The BdM pagodas appear in groups located in mounded areas and their vertical extent is, on average, 70–100 m in Two Way Travel Time (TWTT) (Fig. 7a). Owing to the presence of MS undulations, and taking into account the stratigraphy of the BdM gravity cores^24^, we infer that the formation of the pagoda structures is younger than about 14–12 ka. In addition, as some pagodas intrude and deform the overlying present-day BdM sands (Fig. 7d), the growth of these structures is still active (Fig. 7d).

The occurrence of pagodas not crossing the present-day seabed suggest that (a) the gas upraising and/or the gas-sediment mixing locally stops, and/or (b) the possible, lateral flow of the gas-sediment mixture does not allow local overpressurization processes. According to theoretical models of diapirism^52^, a lateral flow testifies a negative balance between the sediment-gas mixture supply rate from below and the rate of the upward movement of the pagoda. A decrease of the supply rate could be associated with an increase in the density of the mixture due to vanishing gas supply. The above-summarized results, and the buoyancy-controlled uprising of pagodas allow us to estimate the gas column height *h*_*g*_. The buoyancy is given by Δ*P* = *h*_*g*_ *g* (*ρ*_*w*_ – *ρ*_*g*_), where *g* is the gravity (9.8 m/s^2^), and *ρ*_*w*_ and *ρ*_*g*_ are the water and gas densities, respectively. Δ*P* is the sum of the previously calculated *P*_*def*_and the lithostatic pressure *P*_*lith*_ of the sediment plate, which is *ρ*_*s*_ *g h,* with *ρ*_*s*_ the sediment density. In this context, the value of *h*_*g*_ needed for the required buoyancy is given by *h*_*g*_ = (*P*_*def*_ + *P*_*lith*_)/[*g (ρ*_*w*_ – *ρ*_*g*_)]. At BdM, we set *P*_*def*_ = 0.3 Pa and *h* = 100 m (see above), *ρ*_*w*_ = 1,030 kg/m^3^, *ρ*_*s*_ = 2,500 kg/m^3^, negligible *ρ*_*g*_ because *ρ*_*w*_ ≫*ρ*_*g*_. We obtain *h*_*g*_ = 245 m, a value representing the depth of the GSL bottom. Δ*P* is 2.4 MPa and accounts for the overpressure required to break the BdM seafloor and form degassing vents.

The composition of the BdM gases is consistent with a mantle source modified by the addition of fluids related to decarbonation reactions of crustal rocks (Fig. 6). The rough E-W alignment of the BdM dome and the active volcanoes of Ischia, Campi Flegrei, and Somma-Vesuvius, along with the composition of the discharged gases, indicate that the gas released from the mantle beneath the whole Neapolitan volcanic area mixes with increasing amounts of crustal fluids moving from west (Ischia) to east (Somma-Vesuivus) (Figs 1b and 6).

We conclude that a 25 km^2^ wide dome-like structure affected by active degassing processes and resulting from the emplacement of pagodas and mounds occurs in the Gulf of Naples few kilometers offshore from the harbor of Naples. At the present, the BdM features are indicative of a non-magmatic unrest^53^ potentially foregoing embryonic volcanism, i.e. the early emission of magma and/or hot fluids. A monitoring activity should be implemented with the aim to analyze the evolution of the phenomena and detect the geochemical and geophysical signals indicative of a potential magmatic unrest.

## Methods

### Echosounding, ROV and CTD

The acoustic water column profiles (2D) were acquired during the SAFE_2014 (August 2014) cruise performed by the Institute for the Coastal Marine Environment (IAMC) of the National Research Council on board of R/V Urania (CNR). Acoustic sampling was carried out by means of a scientific split-beam echosounders Simrad EK60 working at 38 kHz. Acoustic data were recorded at an average speed of about 4 km. Collected echosounder images were used to identify fluid emissions and accurately define their positions in the acquisition zone (between 74 and 180 m b.s.l.). The physical and chemical parameters in the water column were measured with a multi-parameters probe (Conductivity, Temperature and Depth, CTD). Data were collected by using the CTD 911 probe (SeaBird, Electronics Inc.), and processed with the SBED-Win32 software (Seasave, version 7.23.2). The visual inspection of the seabed was conducted with a ‘Pollux III’ (GEItaliana) ROV equipment (Remote Operated Vehicle) with two (low- and high- definition) video cameras.

### Multibeam data acquisition and processing

Multibeam data acquisition was carried out with a 100 KHz Simrad EM710 multibeam sonar system (Kongsberg). The system was interfaced with a Differential Global Positioning System ensuring a sub-metric error on the beam positioning. The acoustic ping has a 100 KHz frequency, 150° degree for the whole opening of the transmitted pulse, and 400 beams. Sound velocity profiles were measured and real-time applied during the acquisition. Data were processed with the PDS2000 software (Reson-Thales) according to the International Hydrographic Organization standard (https://www.iho.int/iho_pubs/standard/S-44_5E.pdf) for navigation and tidal corrections. Noise reduction due to accidental-instrumental spikes and poor quality beam exclusion was carried out by mean of swath editing and de-spiking tools. Continuous sound velocity probes were carried out by an on-keel station, located near the multibeam transducers, and the sound-speed profiles in the water column were acquired and real-time applied at every 6–8 hours in order to provide the real-time sound speed for a proper beam steering. The whole data set consists of about 440 km^2^ (0–1200 m depth). Data were used to provide a high resolution Digital Terrain Model (DTM) characterized by a 1 m grid cell size. The final DTM (Fig. 1a) is completed by topographic data acquired (at elevation >0 m) by the Italian Geographic Military Institute at 20 m of grid cell size.

### Reflection seismic profiles

An area of about 113 km^2^ was covered with 55 km of high-resolution mono-channel seismic data profiles collected during the Marisk 2007 and Safe 2014 oceanographic cruises, both carried out on-board of the R/V Urania. The Marisk profiles (e.g., the L1 seismic profile, Fig. 1b) were acquired by using an IKB-Seistec boomer system. The acquisition unit consisted of a 2.5 m catamaran where both source and receiver were placed. The source signature consisted of a single positive peak characterized by a 1–10 kHz range of frequencies and allows in resolving reflectors spaced 25 cm apart. Safe seismic profiles were acquired with a 1.4 Kj multi-tip Geospark seismic source interfaced with a Geotrace software (Geo Marine Survey System). This system is made up by a catamaran containing a 1–6.02 KHz seismic source, which has a penetration up to 400 ms in soft sediments below seabed and 30 cm of theoretical vertical resolution. Both the Safe and Marsik equipments were acquired by using a 0.33 shot/sec rate and the vessel velocity was <3 Kn. The data were processed and presented by using the Geosuite Allworks software with the following processing-flow: swell correction, muting of the water column, 2–6 KHz band-pass IIR filtering and AGC.

### Gas sampling and analysis

Gases from the submerged fumarolic vents were collected at the sea bottom using a plastic box, equipped with a rubber septum on its upper side, up-side-down positioned above the vents by the ROV. Once gas bubbles entering the box completely displaced seawater, ROV went back up at 1 m depth where scuba divers transferred through a rubber septum the collected gases into two pre-evacuated 60 mL glass flasks equipped with a Teflon stopcock, one of them being filled with 20 mL of a 5N NaOH solution (Giggenbach-type flasks). The main acidic gas species (CO_2_ and H_2_S) dissolved in the alkaline solution, whereas low-solubility gas species (N_2_, Ar+O_2_, CO, H_2_, He, Ar, CH_4_ and light hydrocarbons) were stored in the sampling flask headspace. Inorganic low-solubility gases were analyzed by gas-chromatography (GC) using a Shimadzu 15A equipped with a 10 m long 5A-molecular sieve column and a thermal conductivity detector (TCD)^54^. Argon and O_2_ were analyzed using a Thermo Focus gas chromatograph equipped with a 30 m long capillary molecular sieve column and a TCD. Methane and light hydrocarbons were analyzed using a Shimadzu 14A gas chromatograph equipped with a 10-m-long stainless steel column packed with Chromosorb PAW 80/100 mesh coated with 23% SP 1700 and a flame ionization detector (FID). The liquid phase was used to analyze 1) CO_2_, as 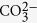, by titration (Metrohm Basic Titrino) with a 0.5 N HCl solution, and 2) H_2_S, as 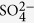, after oxidation with 5 mL H_2_O_2_ (33%) by ion chromatography (IC) (Metrohm 761). The analytical errors for titration, GC and IC analyses were <5%. The analysis of ^13^C/^12^C of CO_2_ (expressed as δ^13^C-CO_2_% vs. V-PDB) was carried out with a Finningan Delta S mass spectrometer after standard extraction and purification procedures of the gas mixtures^55^,^56^. Standards used for estimation of external precision were Carrara and San Vincenzo marbles (Internal), NBS18 and NBS19 (International), whereas the analytical error and the reproducibility were ±0.05% and ±0.1%, respectively.

The δ^15^N (expressed as % vs. Air) values and ^40^Ar/^36^Ar were determined using an Agilent 6890 N gas chromatograph (GC) coupled with a Finnigan Delta plusXP continuous-flow mass spectrometer^57^. Analytical errors were: δ^15^N ± 0.1%, ^36^Ar <1%, ^40^Ar <3%. The He isotopic ratios (expressed as R/Ra, where R is ^3^He/^4^He measured in the sample and Ra is the same ratio in the atmosphere: 1.39 × 10^−6^)^57^ were determined at the laboratory of INGV-Palermo (Italy). ^3^He, ^4^He and ^20^Ne were determined using a double collector mass spectrometer (Helix SFT-GVI)^58^ after separation of He from Ne. The analytical error was ≤0.3%. Typical blanks for He and Ne were <10^−14^ and <10^−16^ mol, respectively.

## Additional Information

**How to cite this article**: Passaro, S. *et al.* Seafloor doming driven by degassing processes unveils sprouting volcanism in coastal areas. *Sci. Rep.* **6**, 22448; doi: 10.1038/srep22448 (2016).

## Supplementary Material

Supplementary Information

Supplementary Video 1

Supplementary Video 2
