# Supplementary material for: Seafloor doming driven by degassing processes unveils sprouting volcanism in coastal areas
Source: Sci Rep. 2016 Mar 1;6:22448. doi: 10.1038/srep22448 (PMC4772541; doi:10.1038/srep22448)
Supplement: Supplementary Information [file srep22448-s1.pdf]

Seafloor doming driven degassing processes unveils sprouting volcanism in coastal areas

Salvatore Passaro[1], Stella Tamburrino[1], Mattia Vallefuoco[1], Franco Tassi[2,3], Orlando Vaselli[2,3], Luciano Giannini[2,3], Giovanni Chiodini[4], Stefano Caliro[5], Marco Sacchi[1], Andrea Luca Rizzo[6], Guido Ventura[1,7]

1 Istituto per l'Ambiente Marino Costiero, Consiglio Nazionale delle Ricerche, Naples, Italy
2 Dipartimento di Scienze della Terra, Università di Firenze, Florence, Italy
3 Istituto di Geoscienze e Georisorse, Consiglio Nazionale delle Ricerche, Florence, Italy
4 Istituto Nazionale di Geofisica e Vulcanologia, Bologna, Italy
5 Istituto Nazionale di Geofisica e Vulcanologia - Osservatorio Vesuviano, Naples, Italy
6 Istituto Nazionale di Geofisica e Vulcanologia, Palermo, Italy
7 Istituto Nazionale di Geofisica e Vulcanologia, Roma, Italy

Supplementary information

Table 1. Gas chemical and isotopic composition in three discharges located in the Gulf of Naples (location in Fig. 1b).

Sample	EM35	EM61	EM52BIS
Date	25/08/14	25/08/14	25/08/14
CO_2	945	934	934
H_2S	0.37	0.44	0.10
O_2	0.62	2.3	3.2
N_2	37	39	43
CH_4	16	24	19
H_2	0.050	0.052	0.027
He	0.015	0.010	0.021
Ne	0.0012	0.0003	0.0005
C_2H_6	0.14096877	0.188935673	0.157879046
C_3H_8	0.024	0.025	0.026
C_3H_6	0.0000022	0.0000024	0.0000023
i-C_4H_{10}	0.0016	0.0018	0.0013
n-C_4H_{10}	0.0025	0.0021	0.0024
i-C_4H_8	0.0058	0.0051	0.0042
C_6H_6	0.059	0.071	0.063
C_7H_8	0.0041	0.0045	0.0043
CS_2	0.0026	0.0029	0.0016
C_4H_4S	0.0011	0.0016	0.0004
C_4H_6S	0.0016	0.0022	0.0011
C_5H_6S	0.0008	0.0013	0.0005
$\delta^{15}N$	1.9830	1.4680	1.7370
^{36}Ar	0.00025	0.00057	0.00061
^{40}Ar	0.078	0.17	0.18
R/Ra	1.94	1.66	1.89
He/Ne	12.85	29.74	43.10
R/Ra c	1.96	1.66	1.90
$\delta^{13}C$-CO_2	-0.93	0.36	-0.44

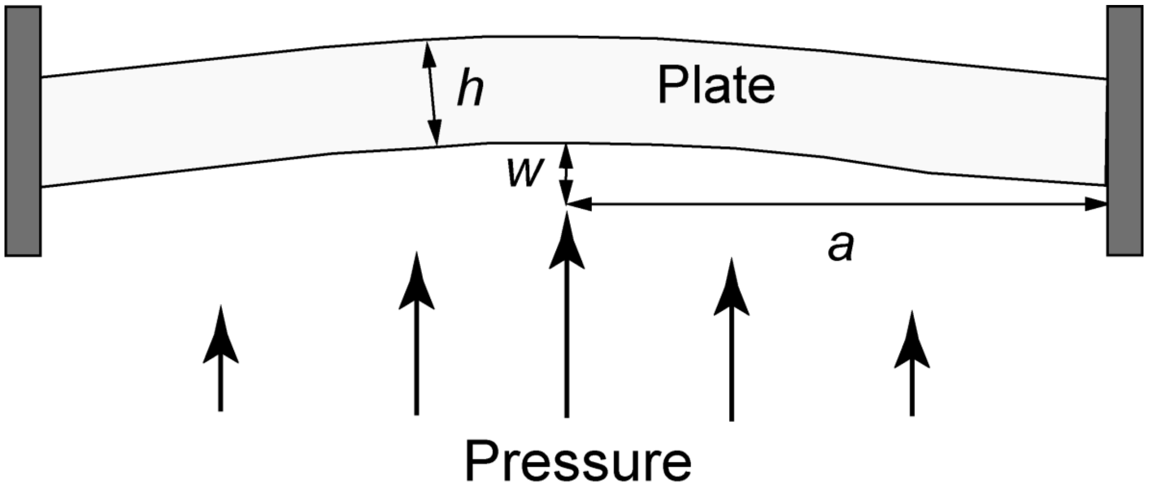

Figure S1. Geometry of a thin-plate bending with fixed boundaries; *w* is the maximum vertical displacement, *a* is radius, and *h* is the layer thickness.

Supplementary Movie 1. Gas discharge at EM_35 (location in Fig.1).

Supplementary Movie 2. Gas sampling with ROV.
